# The Role of Positron Emission Tomography/Computed Tomography in the Management of Differentiated Thyroid Cancer: Current Applications and Future Perspectives

**DOI:** 10.3390/jcm13226918

**Published:** 2024-11-17

**Authors:** Emmanouil Panagiotidis, Jules Tianyu Zhang-Yin

**Affiliations:** 1 Nuclear Medicine, Theageneio Cancer Center, 54639 Thessaloniki, Greece; 2 Department of Nuclear Medicine, Clinique Sud Luxembourg, 486762 Arlon, Belgium

**Keywords:** PET/CT, Thyroid Cancer, 18F-FDG

## Abstract

Differentiated thyroid cancer (DTC), comprising papillary and follicular thyroid carcinoma, is the most common thyroid malignancy and typically has a favourable prognosis when detected early. Positron emission tomography/computed tomography (PET/CT) has emerged as a valuable imaging modality, integrating metabolic and anatomical data. Although PET/CT is not usually part of the initial diagnostic process due to DTC’s indolent nature and low metabolic activity, it plays an essential role in selected clinical scenarios. This includes identifying recurrence in patients with elevated thyroglobulin (Tg) levels and negative radioactive iodine (RAI) scans, evaluating metastatic disease, and guiding treatment in advanced cases. As the use of PET/CT evolves in oncology, this review explores its application in regard to staging, detection of recurrence, and follow-up in terms of managing DTC while also evaluating potential challenges that may occur in the future. The review also considers emerging radiotracers and the theragnostic potential of PET/CT.

## 1. Introduction

Thyroid cancer is the most common endocrine malignancy, with differentiated thyroid cancer (DTC) accounting for more than 90% of cases. DTC includes papillary thyroid carcinoma (PTC) and follicular thyroid carcinoma (FTC), both of which are generally indolent and highly treatable, particularly when diagnosed early. According to the World Health Organization (WHO), the global incidence of thyroid cancer has risen significantly in recent decades, largely due to advances in imaging technology, increased detection of subclinical thyroid nodules, and more widespread use of imaging techniques, including ultrasounds and fine-needle aspiration biopsies (FNAB) [1].

The incidence of thyroid cancer in the United States has significantly increased over the past 40 years, mirroring trends seen globally. It is now the 13th most common cancer overall and the sixth most common among women [2]. The rise is largely attributed to overdiagnosis due to improved diagnostic imaging and tools, particularly for small and localized tumours with high survival rates. However, there are also increasing instances of larger and more advanced thyroid cancers, along with rising mortality rates, suggesting that additional aetiological factors may play a role, albeit to a lesser extent than overdiagnosis.

Historically, childhood exposure to ionizing radiation was the only recognized modifiable risk factor, but obesity has recently emerged as another significant risk factor, despite the unclear biological mechanisms involved. Additionally, recent studies have focused on the impact of endocrine-disrupting chemicals and thyroid dysfunction on cancer development. Advances in identifying molecular subtypes of thyroid cancer and genetic susceptibility factors have also enhanced our understanding of the disease’s etiology. A connection has been established between gut microbiota and thyroid diseases, particularly thyroid cancer. Research indicates that individuals with thyroid cancer often have distinct gut microbiota profiles, suggesting that changes in microbial composition may affect cancer susceptibility and progression [3]. Dysbiosis can trigger inflammatory responses that compromise immune surveillance, potentially facilitating cancer development. Interventions targeting gut microbiota, such as dietary modifications or probiotics, could represent novel therapeutic strategies to enhance immune responses and reduce cancer progression.

While many patients with DTC achieve excellent outcomes, approximately 20% develop recurrence or metastases, underscoring the need for accurate imaging to guide management [4]. Conventional imaging modalities for thyroid cancer include ultrasounds, CT, and radioactive iodine (RAI) scintigraphy. While these techniques are effective in initial diagnosis and follow-up, they may have limitations, particularly in detecting dedifferentiated or non-iodine-avid tumours [5]. Positron emission tomography/computed tomography (PET/CT), particularly using 18F-Fluorodeoxyglucose (FDG) as a tracer, offers both metabolic and anatomical imaging. This hybrid imaging technique has emerged as an essential tool in oncology, particularly in staging, recurrence detection, and monitoring treatment responses in aggressive DTC subtypes [6].

In accordance with the 2015 American Thyroid Association (ATA) guidelines and the 2019 European Thyroid Association (ETA) guidelines for advanced radioiodine refractory thyroid cancer, the primary recommendation for PET/CT in differentiated thyroid carcinoma (DTC) is during follow-up [7,8]. This is particularly relevant for high-risk patients with elevated serum thyroglobulin levels and negative radioiodine imaging, aiding in the detection of residual or recurrent disease when standard imaging fails to provide conclusive results.

Despite its increasing utility, the role of PET/CT in DTC remains a topic of ongoing research. While it is not routinely used in the initial diagnosis of DTC, PET/CT has shown significant value in cases where RAI scintigraphy is inadequate or inconclusive. This review aims to explore the evolving role of PET/CT in managing DTC, focusing on its application in detecting recurrence, staging, and follow-up. In addition, future directions in PET/CT imaging, including the development of novel and theragnostic radiotracers, will be discussed.

## 2. Thyroid Nodules and Diagnosis

Thyroid nodules are common in the general population, with studies indicating a prevalence of up to 68% when assessed using high-resolution ultrasounds. Most of these nodules are benign, with only approximately 5% representing malignancies, most of which are DTC [9]. A fine-needle aspiration biopsy (FNAB) remains the gold standard for the evaluation of thyroid nodules, allowing for cytological analysis and differentiation between benign and malignant nodules [10]. Ultrasound is critical for guiding FNAB and characterizing nodules based on several features, including hypoechogenicity, microcalcifications, irregular margins, and a taller-than-wide shape, all of which are associated with an increased risk of malignancy [10].

However, PET/CT is typically not employed in the initial assessment of thyroid nodules. Most DTCs, especially papillary thyroid carcinomas (PTCs), exhibit relatively low metabolic activity and therefore do not demonstrate high FDG uptake through PET imaging [11]. The American Thyroid Association (ATA) does not recommend routine PET/CT for the initial evaluation of thyroid nodules, as ultrasounds and FNABs are highly reliable in terms of initial diagnostic purposes [7].

**Figure 1:**
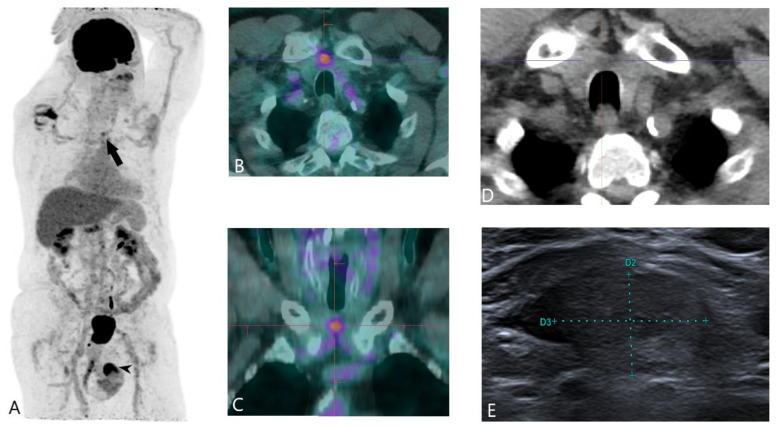
Incidental FDG-avid nodule in the thyroid isthmus discovered during a staging PET/CT study for penile cancer. Fine-needle aspiration confirmed papillary thyroid carcinoma. (**A**) Maximum intensity projection (MIP) of the FDG PET/CT. The black arrow shows a moderate hypermetabolic focus in the thyroid isthmus, and the black arrowhead denotes an intense hypermetabolic focus associated with penile cancer. (**B**) Axial fused and (**C**) coronal fused FDG PET/CT images showing the hypermetabolic nodule in the thyroid isthmus. (**D**) Axial CT image displaying a supra-centimetric, hypodense nodule. (**E**) Ultrasound image showing the nodule measuring 17 × 12 mm.

Nevertheless, PET/CT plays an important role when thyroid nodules are incidentally identified during imaging studies for unrelated malignancies, known as PET incidentalomas. These nodules often demonstrate increased FDG uptake, which raises suspicion for malignancy, particularly in aggressive or dedifferentiated thyroid tumours [12]. It is estimated that approximately 35% of thyroid nodules incidentally detected through PET/CT scans are malignant [12]. Therefore, incidental FDG-avid thyroid nodules warrant further evaluation with an ultrasound and FNAB to exclude the possibility of malignancy (Figure 1).

In a meta-analysis of 34 studies, Treglia et al. analyzed incidental focal thyroid uptake detected by FDG PET/CT in over 200,000 patients, identifying a pooled malignancy risk of around 36% [13]. Another systematic review, which included 18 studies on incidental thyroid uptake (*n* = 55,160), found that most patients (62.1%) had benign conditions while 33.2% had cancer and 4.7% had indeterminate nodules [14]. Among those with cancer, papillary thyroid carcinoma (PTC) was the most prevalent type, making up 82.2% of cases.

FDG PET/CT is also valuable for identifying non-iodine-avid tumours, which are often more aggressive and less differentiated. In these cases, PET/CT can help detect metastatic disease that may not be visualized on traditional RAI scans [15].

## 3. Staging of Differentiated Thyroid Cancer

Staging in DTC is critical for determining prognoses and guiding treatment decisions. The American Joint Committee on Cancer (AJCC) TNM staging system (Table 1) is the most widely used system for evaluating thyroid cancer, incorporating tumour size (T), lymph node involvement (N), and the presence of distant metastasis (M) [7]. Accurate staging is vital, as it directly influences the management approach, including the need for radioactive iodine therapy and the intensity of follow-up. The standard treatment for DTC is a complete thyroidectomy, with or without cervical lymph node dissection based on metastatic risk. In cases of low-risk DTC, a lobectomy may be considered adequate [7].

In most cases, ultrasound and RAI scintigraphy are the primary imaging modalities used for the initial staging of DTC. Ultrasounds are highly sensitive in terms of detecting cervical lymph node metastases, while RAI scintigraphy is effective in identifying distant metastases in iodine-avid tumours [3]. However, in certain cases, these conventional methods may be inadequate, particularly in patients with non-iodine-avid disease or aggressive tumour variants, such as tall cells or Hürthle cell carcinoma [15].

Due to their generally slow growth and low metabolic activity, most well-differentiated thyroid carcinomas exhibit minimal FDG uptake. Consequently, the primary role of FDG PET/CT in DTC management is typically limited to postoperative follow-up. Given the low incidence of initial distant metastasis (4–7%), routine initial staging with PET is often not indicated [16,17,18]. However, the emerging literature suggests that PET/CT may have potential utility in identifying recurrence or metastasis in high-risk groups or patients with atypical presentations. This indicates a need for further research to better understand when PET/CT might be beneficial in the diagnostic pathway for DTC, especially in more complex or advanced cases.

The “flip-flop phenomenon” refers to the inverse relationship between the radioiodine uptake and the metabolic activity (FDG) in thyroid cancer, where tumours that are iodine-negative may demonstrate an increased FDG uptake [19]. This phenomenon highlights the importance of using FDG PET/CT in cases where traditional iodine imaging fails to identify malignant lesions, particularly in aggressive subtypes of differentiated thyroid carcinoma.

Although PET/CT may offer potential benefits in staging thyroid cancer, current ATA guidelines indicate a lack of strong evidence or consensus to support its routine use as a pre-operative tool, regardless of tumour differentiation or metastatic status [7].

## 4. Recurrence Detection in Differentiated Thyroid Cancer

**Figure 2:**
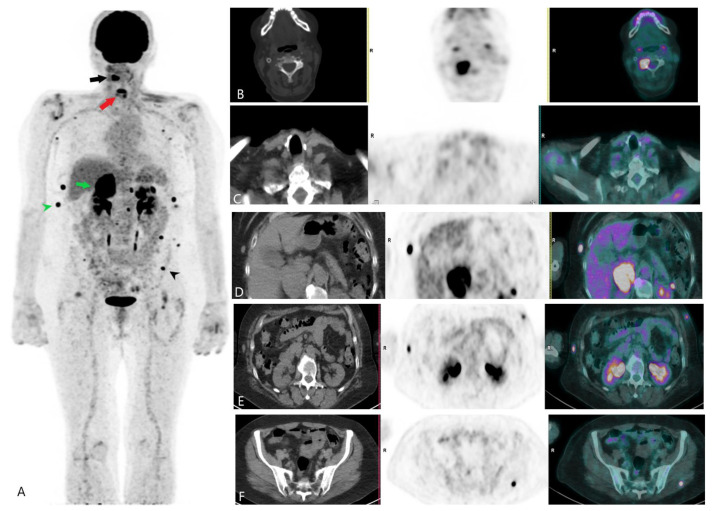
FDG PET/CT scan assessing multi-metastatic recurrence of iodine-refractory papillary thyroid carcinoma before treatment with tyrosine kinase inhibitors. (**A**) Maximum intensity projection (MIP) of the FDG PET/CT. The black arrow indicates an intensely FDG-avid bone metastasis in the right posterior arch of the C3 vertebra. The red arrow marks the site of total thyroidectomy. The green arrow shows FDG-avid foci in a right adrenal gland metastasis. The green arrowhead points to the right lateral thoracic subcutaneous metastasis. The black arrowhead indicates an FDG-avid left gluteus maximus deposit. (**B**) Axial CT, PET, and fused PET/CT images displaying intense hypermetabolic focus in the right posterior arch of C3. (**C**) Axial CT, PET, and fused PET/CT images depicting the site of total thyroidectomy. (**D**) Axial CT, PET, and fused PET/CT images showing the FDG-avid right adrenal gland metastasis. (**E**) Axial CT, PET, and fused PET/CT images illustrating hypermetabolic subcutaneous metastasis of the right thoracic region laterally. (**F**) Axial CT, PET, and fused PET/CT images revealing FDG-avid left gluteus maximus deposit.

**Figure 3:**
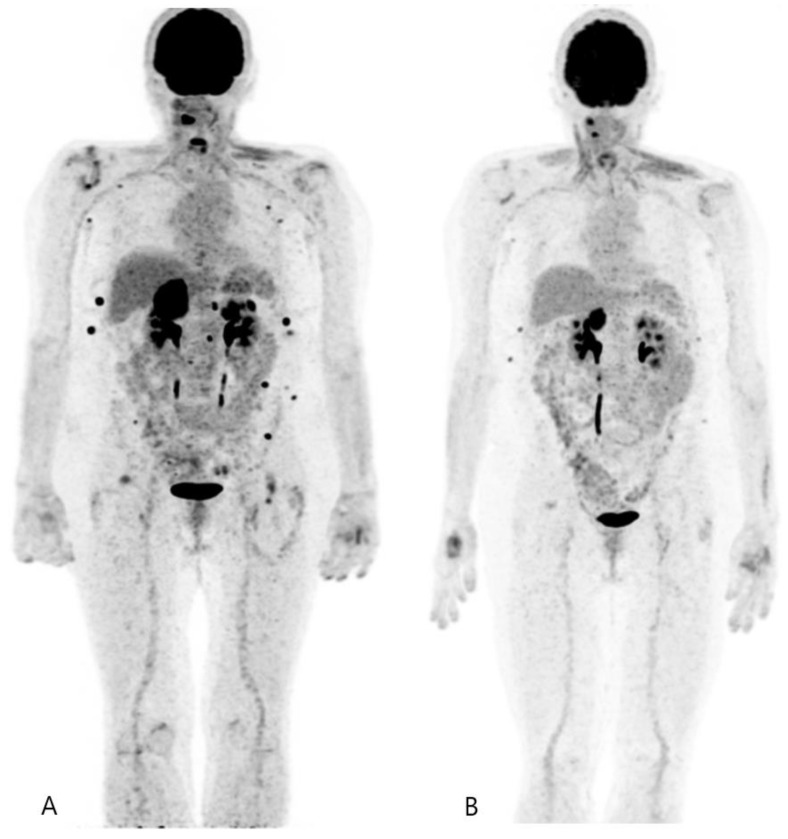
FDG PET/CT scans evaluating the response of multi-metastatic iodine-refractory papillary thyroid carcinoma following treatment with tyrosine kinase inhibitors. (**A**) MIP image obtained before treatment. (**B**) MIP image three months post-treatment with tyrosine kinase inhibitors, showing a partial response.

A significant challenge in the long-term management of DTC patients is identifying recurrence, which occurs in approximately 20–30% of cases, often years after the initial treatment [20]. While DTC generally has a favourable prognosis, specific subgroups experience diverse outcomes. For example, young patients with small tumours often have excellent long-term survival, while older patients with aggressive histology, extensive lymph node involvement, or distant metastases face a more guarded prognosis (Figure 2 and Figure 3). Nevertheless, early detection of recurrent disease is crucial, particularly for patients with aggressive disease subtypes or those who develop distant metastases. Thyroglobulin (Tg) serves as a reliable biomarker for DTC recurrence, with elevated levels often preceding imaging findings [20]. However, in a subset of patients with elevated Tg and negative RAI scans, the disease can be difficult to localize.

FDG PET/CT has proven to be a powerful tool in identifying sites of recurrence, particularly in patients with elevated Tg levels and negative RAI scans. FDG PET/CT detects non-iodine-avid metastatic disease, often undetectable by traditional RAI scans, by targeting cells with high metabolic activity, such as dedifferentiated thyroid cancer cells that have lost their ability to uptake iodine [20]. A study investigated the relationship between Tg kinetics (doubling time and velocity) and metabolic activity in DTC patients [21]. The results showed that patients with higher Tg levels and faster Tg kinetics were more likely to have positive PET/CT scans. Additionally, Tg kinetics were found to be independent prognostic factors for overall survival.

Moreover, PET/CT has demonstrated clinical utility in patients with biochemically incomplete responses to initial treatment (i.e., persistently elevated Tg levels post-surgery and RAI therapy) but no evidence of disease on conventional imaging. This patient cohort is at increased risk for recurrence, and early identification of metastatic or recurrent disease with PET/CT can significantly alter the treatment approach. For instance, it can guide decisions regarding re-surgery, targeted therapy, or external beam radiation [21].

Another study investigated the long-term prognostic value of FDG PET/CT in patients with differentiated thyroid cancer undergoing empiric RAI therapy [22]. The researchers found that FDG PET/CT was more effective than post-therapy WBS in predicting disease recurrence. Patients with negative FDG PET/CT and normalized Tg levels had a better prognosis, with higher rates of disease-free and overall survival. These findings suggest that FDG PET/CT can be a valuable tool for risk stratification in this patient population.

Cross-sectional imaging (MRI and CT) may be useful for prognostic assessment in patients with metastatic disease or those at high risk of rapid progression, while PET/CT is often preferred for evaluating response to systemic or local therapies after treatment. However, the decision between conventional imaging and PET/CT for this purpose remains a subject of debate [7]. The ETA guidelines suggest that PET/CT can provide additional insights into tumour biology; however, their prognostic or management implications are yet to be fully established [8].

In addition to high-risk patients with elevated Tg and negative RAI imaging, PET/CT may also be recommended for patients with suspected recurrence and in cases with aggressive histological subtypes (e.g., tall cell, Hürthle cell, or poorly differentiated variants) of DTC. PET/CT can be particularly valuable in identifying metastatic disease in these patients when conventional imaging is inconclusive, aligning with current recommendations and clinical practice guidelines for the management of high-risk or recurrent DTC [7,8]. While a TSH-stimulated Tg level of 10 ng/mL is often used as a cut-off, this may need to be adjusted in cases of aggressive thyroid cancer subtypes [23]. Albano et al., explored the effectiveness of Tg doubling time (Tg-DT) versus absolute Tg levels in identifying patients with non-iodine avid DTC who may benefit from FDG PET/CT imaging [24]. They found that Tg-DT offered a more reliable threshold than Tg levels alone in identifying patients likely to benefit from PET/CT. Faster Tg-DT was associated with a higher likelihood of detecting metastases, suggesting its potential to guide personalized treatment strategies. The study highlights the prognostic value of Tg kinetics over static Tg levels in managing DTC patients with negative radioiodine scans.

Dong et al. reviewed 25 studies with a total of 789 patients and found that FDG PET/CT exhibited a high pooled sensitivity of 93.5% for identifying recurrence and metastasis of DTC in instances without RAI uptake [25]. Similarly, Miller et al. conducted a meta-analysis of 12 studies which revealed a sensitivity of 94.0% for PET/CT in detecting recurrences of PTC [26]. Compared to conventional imaging methods, PET/CT was more effective in identifying recurrent or metastatic DTC. Weber et al. reported that ultrasounds detected recurrent or metastatic thyroid disease in only 57% of cases, whereas Seo et al. demonstrated that 21.1% of lymph node and soft-tissue lesions missed by neck ultrasounds were recognized by PET/CT [27,28,29]. Additionally, PET/CT outperformed PET alone in detecting small metastatic lesions.

Giovanella et al. found that 88% of patients (*n* = 102) with a positive FDG PET/CT scan had Tg levels greater than 5.5 ng/mL [30]. Notably, dedifferentiated thyroid carcinoma cells may have a reduced capacity to produce and secrete Tg, indicating that a low Tg level in patients with a negative 131I scan does not necessarily reflect a minimal tumour burden. While the impact of thyroid-stimulating hormone (TSH) levels on radioiodine scans is well recognized, there is currently no agreement on TSHs’ influence on the accuracy of FDG PET/CT. It has been proposed that TSH suppression may be advantageous for patients with low Tg levels (< 10 ng/mL) who adhere to hypothyroidism management, whereas TSH stimulation using recombinant TSH should be considered for those unable to tolerate hypothyroid symptoms [20].

## 5. Follow-Up in Differentiated Thyroid Cancer

The American Thyroid Association (ATA) guidelines recommend periodic follow-up for DTC patients based on risk stratification, with imaging primarily using ultrasound and RAI scans in low- to intermediate-risk patients [7]. However, patients with high-risk DTC, particularly those with persistent disease after initial treatment or aggressive histologic variants, may require more intensive surveillance. PET/CT is increasingly recognized as a valuable tool in these cases due to its ability to detect early recurrence and non-iodine-avid metastases [31].

A study highlighted the significant role of PET/CT in patients with suspicious RAI scans or aggressive tumour variants [32]. FDG PET/CT proved to be a valuable tool for monitoring high-risk thyroid carcinoma, particularly in patients whose post-therapeutic 131I whole-body scans were inconclusive or not proportional to stimulated Tg levels, as well as in those with aggressive DTC variants. Furthermore, the study demonstrated that FDG PET/CT was linked to disease progression and effectively revealed undifferentiated lesions, aiding clinical evaluations regarding surgical interventions or watchful waiting strategies.

When used alongside Tg, FDG PET/CT provides important prognostic information and is crucial for informing clinical decisions in patients with DTC who have negative 131I scans. Vural et al. noted a higher prevalence of PET positivity in patients over 40 years compared to younger individuals (70% vs. 53%) [33]. Given that the age at which distant metastasis is detected is independently associated with mortality, and that PET positivity is more frequent in older patients, it can be inferred that FDG uptake may be linked to a worse prognosis and more aggressive tumour behaviour [34].

Robbins et al. examined the prognostic significance of metabolic activity in metastatic thyroid carcinoma among 400 reviewed patients [35]. Results showed that age and FDG-PET findings remained strong predictors of survival in multivariate analysis while the initial AJCC stage was insignificant. Patients with negative FDG PET scans had a 2-year survival rate of 99%, while those with the highest Standardized Uptake Value maximum (SUVmax) had a significantly lower rate of 52%, suggesting that FDG PET can effectively stratify patients into low- and high-risk groups for cancer-related mortality. The authors concluded that FDG PET scanning effectively stratifies patients into low-risk (FDG-negative) or high-risk (FDG-positive) categories for cancer-associated mortality, suggesting that treatment aggressiveness should correspond to FDG PET status.

Piao et al. studied the combined use of metastatic lymph node ratio (LNR) and Tg levels to determine the need for FDG PET in detecting persistent disease in patients with PTC [36]. The study included 429 PTC patients who underwent surgery and RAI therapy, with evaluations being performed prior to RAI therapy. Key cut-off values were established for serum Tg (6.0 ng/mL), the number of metastatic lymph nodes (5), and LNR (0.51) to guide PET/CT indications. The LNR-combined criteria demonstrated significantly better diagnostic performance in identifying FDG-avid persistent disease compared to using individual parameters. This approach offers a more tailored strategy for utilizing PET/CT in the management of PTC patients, potentially improving the detection of persistent disease.

The study by Gim et al. evaluated the diagnostic value of SUV in FDG PET/CT for PTC, particularly focusing on the role of BRAF mutations [37]. They found that higher SUV values were significantly associated with the presence of BRAF V600E mutations, indicating that metabolic activity, as measured by PET/CT, could reflect tumour aggressiveness. Additionally, the study highlighted how those patients with BRAF-mutated tumours tended to exhibit increased FDG uptake, suggesting a potential for using SUV metrics as a prognostic marker. Overall, the findings underscore the importance of incorporating BRAF status into PET imaging assessments for improved management of PTC.

A systematic review and meta-analysis examined the correlation between BRAF V600E mutation status and FDG PET/CT activity in PTC [38]. The analysis included 12 studies and found that patients with the BRAF V600E mutation had a significantly higher likelihood of exhibiting FDG-avid lesions, with a pooled odds ratio of 2.12. Additionally, the study found a substantial difference in SUVmean between BRAFV600E-positive and BRAFV600E-negative patients, with the former group showing an average increase of 5.1. These findings indicate that the presence of the BRAF V600E mutation is associated with increased metabolic activity, underscoring its relevance in assessing disease aggressiveness and recurrence risk in PTC.

Emerging studies suggest that FDG PET/CT could play an even more significant role in risk stratification during follow-up, particularly in patients with advanced disease. In active surveillance, PET/CT is used to monitor patients undergoing systemic therapy, such as tyrosine kinase inhibitors (TKI) therapy or immune checkpoint inhibitors [20]. These therapies are primarily used in patients with radioactive iodine-refractory DTC (RAIR-DTC), where disease progression can be unpredictable, and conventional imaging modalities may fail to capture the full extent of the disease [14]. PET/CT has proven to be a sensitive modality for detecting subtle changes in metabolic activity, providing a reliable method for assessing therapeutic responses in patients undergoing these treatments [15].

In conclusion, while FDG PET/CT shows promise in various scenarios for differentiated thyroid cancer, clear guidelines are still lacking. Consequently, its clinical use may vary significantly among different medical centres.

### 5.1. Emerging Role of Novel Radiotracers (Table 2)

While FDG PET/CT remains the gold standard for detecting dedifferentiated thyroid cancer, the development of novel radiotracers offers the potential for improved sensitivity and specificity.

**Table 2:** Radiotracers for PET/CT in Thyroid Cancer.

Radiotracer	Target	Applications
18F-FDG	Glucose metabolism	Detecting non-iodine-avid, aggressive, or recurrent DTC; monitoring treatment response
124I	Sodium iodide symporter	Pre-therapeutic dosimetry; high-resolution imaging for iodine-avid DTC metastases
18F-FDOPA	Amino acid transport	Imaging for DTC with neuroendocrine features or medullary thyroid carcinoma
68Ga-DOTA-TATE/NOC/TOC	Somatostatin receptors	Used in imaging neuroendocrine DTC variants and medullary thyroid carcinoma
18F-TFB	Sodium iodide symporter (NIS)	Non-invasive NIS imaging, recurrence detection, pre-operative evaluation
68Ga-DOTA-FAPI-04	Fibroblast activation protein	Detection of RAI-refractory DTC lesions

One promising alternative is 18F-dihydroxyphenylalanine (FDOPA), which is particularly relevant in the context of the neuroendocrine features of thyroid cancers, such as medullary thyroid carcinoma (MTC) [39]. FDOPA PET/CT exploits the uptake of this radiotracer by dopamine receptors, which are often expressed in higher levels in certain thyroid malignancies compared to the glucose transporters used in FDG imaging.

Additionally, Somatostatin receptor-targeting radiopharmaceuticals are utilized for imaging tumours demonstrating high levels of somatostatin receptors (SSTRs). The most common 68Ga-labelled somatostatin analogues, [68Ga]Ga-DOTA-TATE, [68Ga]Ga-DOTA-NOC, and [68Ga]Ga-DOTA-TOC, bind to different somatostatin receptor (SSTR) subtypes. [68Ga]Ga-DOTA-TATE targets SSTR2; [68Ga]Ga-DOTA-NOC targets SSTR2 and SSTR3; and [68Ga]Ga-DOTA-TOC targets SSTR2 and SSTR5. Notably, differentiated thyroid carcinoma cells often overexpress SSTR2, SSTR3, and SSTR5. Additionally, 68Ga-DOTATATE PET/CT, which targets somatostatin receptors, is being investigated for its potential to detect neuroendocrine features in thyroid cancer variants, such as medullary thyroid carcinoma (MTC) and poorly differentiated thyroid cancer [40]. This radiotracer has demonstrated high sensitivity in detecting somatostatin receptor-positive tumours and may offer a new avenue for imaging DTC variants that are poorly visualized in regard to FDG PET/CT [41].

Radiolabelled somatostatin analogues can potentially detect DTC recurrence or metastases, particularly in patients with RAI-refractory disease. Ocak et al. examined 13 patients with RAIR-DTC (nine with PTC, one with FTC, and three with Hurthle cell carcinoma) to compare the effectiveness of [68Ga] Ga-DOTA-TATE and [68Ga] Ga-DOTA-NOC in detecting metastatic lesions [42]. Somatostatin-positive lesions were identified in eight (62%) pts, [68Ga] Ga-DOTA-TATE was detected in 45 lesions, and [68Ga] Ga-DOTA-NOC was detected in 42 lesions. The uptake of lesions was significantly greater with [68Ga] Ga-DOTA-TATE (SUVmax 12.9 ± 9.1) compared to [68Ga] Ga-DOTA-NOC (SUVmax 6.3 ± 4.1), indicating the former’s potential superiority in imaging RAIR-DTC. The detection of positive RAIR-DTC lesions through somatostatin receptor imaging paves the way for treating these patients with peptide receptor radionuclide therapy (PRRT), a targeted therapeutic strategy.

In 2017, a novel PET tracer, 18F-tetrafluoroborate (18F-TFB), was tested in healthy human subjects for imaging DTC. This study demonstrated promising results for non-invasive NIS (sodium iodide symporter) imaging, as 18F-TFB can mimic iodide transport, entering thyroid cells through the NIS receptor. Additionally, 18F-TFB has diagnostic potential in detecting recurrent DTC and cervical lymph node metastases. It can be used to identify pre-operative cervical lymph node metastases, avoiding unnecessary surgery, and to predict the success of radioiodine therapy [20]. Notably, 18F-TFB PET/CT offers three key advantages over 123I or 131I scintigraphy:

Higher sensitivity: As a positron emitter detected by PET, 18F-TFB provides greater sensitivity compared to gamma camera-based scintigraphy [43].

Same-day imaging: Unlike iodine-123 (131) scintigraphy, 18F-TFB PET/CT can be performed on the same day as tracer administration, reducing patient inconvenience.

Lower radiation dose: 18F-TFB delivers a lower radiation dose to iodine-123 (131) scintigraphy.

Fibroblast activation protein (FAP) expression is significantly elevated in cancer-related fibroblasts compared to normal fibroblasts. Due to their abundance in many tumours, radiolabelled fibroblast activation protein inhibitors (FAPIs) show promise for tumour imaging. Chen et al. assessed the effectiveness of [68Ga] Ga-DOTA-FAPI-04 PET/CT in detecting RAIR-DTC lesions [44]. Among 24 patients with RAIR-DTC, 21 (87.5%) had FAPI-positive lesions, with a mean SUVmax of 4.25 and a growth rate of 6.51%. Additionally, SUVmax showed a positive correlation with lesion growth rates.

The development of theragnostic radiotracers, such as 131I-labelled tracers, which are both diagnostic and therapeutic, represents a promising frontier in thyroid cancer management. The concept of theragnostic radiotracers involves using PET/CT to identify tumours that express specific molecular targets (e.g., somatostatin receptors) and then deliver targeted radiotherapy to these tumours. This approach is currently being tested in clinical trials for RAIR-DTC, offering the potential for more personalized treatment strategies [45].

### 5.2. Role of 124I PET/CT in Differentiated Thyroid Cancer

Iodine-124 (124I) is an isotope used in PET imaging which, unlike 131I, allows for higher-resolution imaging due to the superior sensitivity and spatial resolution of PET technology compared to gamma scintigraphy or SPECT (Single Photon Emission Computed Tomography). Notably, 124I PET/CT is particularly useful in pre-therapeutic settings for patients undergoing RAI therapy with 131I, as it allows for individualized dosimetry and improved detection of iodine-avid lesions [46].

The high sensitivity of 124I PET/CT for detecting smaller metastases, particularly in lymph nodes and the lungs, underscores its effectiveness in identifying recurrent DTC. This enhanced sensitivity is vital for early intervention, as timely detection of metastases can significantly influence prognosis and treatment outcomes. Although 124I PET/CT may not be used in every case, its potential as a crucial diagnostic tool is especially evident in select patient populations, such as those at high risk of recurrence or who present with atypical features.

### 5.3. Advantages of 124I PET/CT

Pre-therapeutic Dosimetry: One of the most significant advantages of 124I PET/CT is its ability to provide personalized dosimetry. By using 124I PET imaging before administering therapeutic doses of 131I clinicians can accurately measure the iodine uptake in both the primary tumour and metastatic sites, allowing for the calculation of optimal therapeutic doses. This is particularly valuable in patients with RAIR-DTC or those at risk of over- or under-treatment with standard 131I dosages [47]. Also, 124I PET allows for precise quantification of RAI uptake in metastatic sites, which can significantly impact treatment decisions. For example, it can guide the decision to escalate or de-escalate the dose of 131I or determine whether RAI therapy is even appropriate for patients with low uptake [48].

High Sensitivity for Metastatic Disease: In contrast to conventional 131I imaging, 124I PET/CT provides a higher resolution, enabling the detection of smaller metastases and more precise localization of iodine-avid lesions. Studies have shown that 124I PET/CT can detect micro-metastases, particularly in lymph nodes and pulmonary metastases, which might be missed by SPECT/CT with 131I [48].

Combination with FDG PET/CT: In certain cases, a combined approach using 124I PET/CT and FDG PET/CT may be employed to evaluate both iodine-avid and non-iodine-avid metastatic sites, particularly in patients with advanced, aggressive variants of DTC. This dual imaging strategy provides a more comprehensive view of the disease burden, especially for patients with heterogeneous metastatic disease (i.e., both iodine-avid and non-avid lesions) [49].

Assessment of RAIR-DTC: One critical application of 124I PET/CT is evaluating the extent of disease and functional status of metastatic lesions in RAIR-DTC patients. By accurately assessing iodine uptake, 124I PET/CT helps identify patients who are unlikely to benefit from further RAI therapy and may need alternative treatments, such as TKIs or external beam radiotherapy [50].

Notably, 124I PET/CT is increasingly utilized in staging and treatment planning for patients with extensive metastatic thyroid cancer. By assessing iodine uptake in metastatic sites, 124I PET/CT enables clinicians to tailor RAI therapy based on tumour-specific dosimetry. This personalized approach can improve treatment outcomes by increasing the likelihood of a complete response and reducing the risk of radiation-induced toxicity. Additionally, 124I PET/CT can identify non-iodine-avid metastases, guiding the selection of alternative treatments like surgery, external radiation, or systemic therapies. Moreover, by distinguishing metabolically active tumours from non-functional tissue, 124I PET/CT can aid in differentiating active metastatic disease from benign or fibrotic changes post-treatment. Several studies have demonstrated the effectiveness of 124I PET/CT in optimizing RAI therapy dosing and improving patient outcomes [51].

### 5.4. Emerging Applications of 124I PET/CT

Recent research explores the use of 124I PET/CT in combination with other molecular imaging techniques and novel tracers. Theragnostic use of 124I-labelled molecules offers a glimpse into the future of personalized thyroid cancer therapy. By combining PET imaging with targeted radioisotope therapy, clinicians may be able to treat specific tumour sites while sparing healthy tissue, a key goal in advancing the management of RAIR-DTC.

Despite its numerous advantages, there are some limitations to 124I PET/CT:

Availability and Cost: The use of 124I is limited by its high cost and the need for specialized equipment and expertise in dosimetry calculations. These factors make it less accessible in resource-constrained settings [51].

Radioactive Half-Life: Notably, 124I has a shorter half-life (approximately 4 days) to 131I, meaning that the window for imaging is shorter and patients may require repeated scans to accurately measure iodine kinetics and calculate dosimetry.

Radiation Dose: Although 124I provides high-resolution imaging, it also delivers a higher radiation dose to patients compared to diagnostic doses of 131I, which may limit its use, particularly in pediatric or low-risk patients [50].

## 6. Future Challenges and Perspectives

Advances in molecular imaging, particularly the development of novel radiotracers that target specific molecular pathways, offer significant promise in overcoming current limitations in thyroid cancer management. For instance, 124I PET/CT has shown considerable potential in improving the detection of iodine-avid metastatic disease, particularly in RAIR-DTC patients. This imaging modality provides more accurate dosimetry and enables the visualization of smaller metastases that may be missed by traditional RAI scans. Additionally, theragnostic approaches, which combine diagnostic imaging with targeted radionuclide therapy, are emerging as a highly personalized treatment strategy for advanced DTC. This dual-function approach could further refine the role of PET/CT in treatment planning, offering the ability to both detect disease and deliver therapy based on molecular characteristics, such as somatostatin receptor expression or glucose transporter (GLUT) upregulation.

Another significant challenge lies in the integration of PET/CT into the current clinical management guidelines for DTC. While PET/CT is increasingly used in specific clinical contexts, such as in cases of elevated Tg levels but negative conventional imaging, its role remains largely supplementary to established imaging methods, such as ultrasounds and conventional RAI scintigraphy. Further research and large-scale studies are required to establish standardized criteria for the optimal use of PET/CT in DTC management. Studies like the THYROPET study have already highlighted the potential of combining 124I PET/CT and FDG PET/CT in preventing futile RAI therapies [49]. Moreover, transcriptomic analysis in papillary thyroid cancer (PTC) patients, as shown in recent studies, indicates that tumour size and metabolic activity (SUVmax) are associated with distinct gene expression profiles, providing a potential avenue for PET/CT to be used in more personalized treatment approaches [52].

Future challenges in managing DTC involve integrating molecular insights from SUVmax and gene expression profiles into clinical practice. SUVmax is associated with tumour aggressiveness, glucose metabolism, and pathways related to DNA replication, with distinct molecular characteristics being observed in high SUVmax tumours. Based on the subgroup analysis, genes like PSG5, TFF3, SOX2, SL5A5, SLC5A7, HOXD10, FER1L6, and IFNA1 were significantly associated with tumour aggressiveness [53]. Both high SUVmax papillary thyroid microcarcinoma (PTMC/a thyroid cancer with small tumours that are 1.0 cm maximum in diameter) and macro-PTC are enriched in the pathways of DNA replication and cell cycles, while gene sets for purine metabolism are enriched only in high SUVmax macro-PTC. These findings highlight the molecular characteristics of high SUVmax tumours and the metabolism involved in DTC growth. However, challenges remain in standardizing advanced imaging in terms of FDG PET/CT and in identifying patients who would benefit most. Further research and advances in molecular imaging and theragnostics are crucial in regard to refining personalized treatment strategies for aggressive DTC.

The expanded discussion on transcriptomic analysis in DTC management highlights its potential to guide treatment decisions and enhance patient stratification. Recent studies have identified specific gene expression profiles that correlate with tumour aggressiveness, opening new avenues for personalized management strategies [53]. By understanding the molecular characteristics of tumours, clinicians can better categorize patients according to their risk levels, allowing for tailored treatment approaches that improve outcomes for those with high-risk or aggressive forms of DTC. Moreover, transcriptomic profiling enhances the ability to predict recurrence risk, facilitating more effective follow-up protocols. Patients identified as having a higher likelihood of recurrence can benefit from closer monitoring and timely interventions, while those at lower risk may require less frequent follow-ups. This personalized approach optimizes patient care and minimizes unnecessary procedures and healthcare costs. As research in this area continues to advance, integrating transcriptomic data into clinical practice will be crucial for improving DTC management and patient outcomes [54].

Future perspectives should also focus on improving access to novel imaging technologies, such as [68Ga] Ga-DOTA-based PET/CT tracers, which are currently used for imaging somatostatin receptor-positive neuroendocrine tumours and may also be beneficial in select cases of DTC with neuroendocrine differentiation. As molecular imaging continues to evolve, its integration into routine clinical practice for DTC will depend on robust evidence that demonstrates improved patient outcomes and cost-effectiveness.

Prostate-specific membrane antigen (PSMA), a transmembrane protein encoded by the FOLH1 gene, has emerged as a promising target for molecular imaging and therapy in prostate cancer. However, recent studies have shown that PSMA is also frequently expressed on the cell membranes of neovascular endothelial cells in various solid tumours beyond prostate cancer. This broader expression suggests that PSMA-targeted PET imaging could serve as a diagnostic tool for other tumour types, including thyroid cancer [55].

While studies suggest that PSMA PET/CT may outperform FDG PET/CT in detecting bone and lung lesions, and that FAPI-based tracers show promise in identifying radioiodine-refractory disease, their clinical utility must be validated through larger trials [45]. Moreover, 18F-TFB’s potential for non-invasive NIS imaging presents a valuable opportunity for improved cervical lymph node metastasis detection. However, the limitations of the current literature—such as small sample sizes, variability in study designs, and the potential omission of relevant studies—underscore the challenge of drawing definitive conclusions. The observed heterogeneity among studies further complicates the establishment of clear guidelines for clinical application. Therefore, future research should prioritize head-to-head comparisons of these tracers to enhance diagnostic accuracy and optimize patient stratification in the evolving landscape of DTC management.

Advances in molecular imaging, such as the development of novel radiotracers that target specific molecular pathways in thyroid cancer, may overcome some of these limitations. For example, iodine-124 PET/CT has shown promise in improving the detection of iodine-avid metastatic disease, offering a potential alternative to traditional RAI scans. Additionally, theragnostics, which combines diagnostic imaging and targeted therapy, is an emerging field that could further refine the role of PET/CT in the management of DTC.

Another challenge is the integration of PET/CT into the current management guidelines for DTC. While PET/CT is increasingly used in certain clinical situations, its role remains largely supplementary to conventional imaging methods such as ultrasounds and RAI scanning. Further research is needed to establish standardized criteria for its use and to determine which patients are most likely to benefit from PET/CT.

Positron Emission Tomography/Magnetic Resonance Imaging (PET/MRI) is a hybrid imaging technology that combines the metabolic imaging capabilities of PET with the superior soft-tissue contrast of MRI. Unlike PET/CT, which exposes patients to significant radiation from both PET tracers and CT scans, PET/MRI reduces radiation exposure by replacing CT with MRI. Additionally, MRI provides excellent anatomical detail and functional imaging, which is particularly advantageous in the neck region, a challenging anatomical area with complex soft-tissue structures where lymph nodes and soft tissues are closely adjacent, as in thyroid cancer cases. A study that assessed the diagnostic efficacy of PET/MRI versus PET/CT in patients with differentiated thyroid carcinoma using the iodine-124 radiotracer found that PET/MRI of the neck demonstrated superior sensitivity compared to PET/CT in detecting iodine-positive lesions, attributed primarily to the enhanced sensitivity of the PET component [56]. However, while PET/MRI showed advantages in some cases, it did not provide a significant benefit over PET/CT for differentiating iodine-positive lesions as either metastases or thyroid remnants. The volumetric data provided by MRI for certain iodine-positive lesions may, nonetheless, offer useful information for dosimetry purposes.

## 7. Limitations

While PET/CT is a useful tool in diagnosing thyroid cancer, it does come with certain limitations. One significant issue is the use of iodinated contrast agents in CT scans, which can impact the accuracy of subsequent radioiodine scans. These contrast agents may remain in the body for up to six weeks, potentially interfering with the uptake of radioactive iodine needed for assessing treatment response and recurrence in thyroid cancer [57].

Another drawback of PET/CT is the potential for false positives when using the 18F-FDG tracer. This tracer highlights areas of high metabolic activity which can occur in both cancerous and benign conditions, such as infections or inflammation, complicating the distinction between residual cancer and post-treatment inflammation in the thyroid region. PET/CT also faces challenges in detecting small lung metastases due to its thicker image slices; in such cases, high-resolution CT is often preferred [58]. Additionally, PET/CT offers limited resolution for assessing potential metastases in the brain or spine, whereas MRI or PET/MRI provide superior imaging details.

## 8. Cost-Effectiveness

The cost-effectiveness of PET/CT in managing DTC has become a pivotal aspect of optimizing patient care and healthcare resources. Traditional imaging methods often struggle to accurately detect recurrent or metastatic DTC, which can result in unnecessary interventions and increased healthcare expenditures. Integrating PET/CT into standard diagnostic protocols enhances the detection of non-iodine metastatic lesions, enabling clinicians to tailor treatment plans more effectively. Although the initial costs of FDG PET/CT may exceed those of conventional imaging, studies, including those by Vogel et al., demonstrate that the long-term savings achieved through improved patient outcomes and reduced rates of ineffective treatments can justify its implementation, particularly in high-risk patient populations [59].

Moreover, the precision of FDG PET/CT in staging and assessing disease progression supports better decision-making in treatment planning. Patients with a higher risk of recurrence benefit from more intensive monitoring and timely interventions, ultimately leading to improved survival rates and quality of life. The cost-effectiveness of incorporating FDG PET/CT into clinical routines highlights its potential not only to enhance diagnostic accuracy but also to promote overall healthcare efficiency by minimizing unnecessary healthcare costs. As evidence supporting its role in DTC management continues to accumulate, Vogel et al. reinforce the argument for the routine use of FDG PET/CT, emphasizing its promise in optimizing resource allocation while improving patient outcomes in the long term [59].

### Endocrine Societies’ Perspectives

The American Thyroid Association (ATA) and the European Thyroid Association (ETA) have reached a consensus on the selective application of FDG PET/CT in DTC, underscoring its use in specific high-risk scenarios while advising against routine implementation [7,10].

## 9. Recommendations

### 9.1. Supplementary Tool for Recurrence Detection

The ATA recommends FDG PET/CT primarily as a supplementary diagnostic tool for detecting recurrence in DTC, particularly in cases where serum Tg levels are elevated but RAI scans are negative.

It is not endorsed for initial diagnosis due to DTC’s generally low FDG uptake during the early stages.

### 9.2. High-Risk Monitoring

For patients with suspected recurrence (elevated Tg levels) and inconclusive conventional imaging (e.g., RAI scintigraphy, CT, MRI or ultrasound), FDG PET/CT is suggested.

The ATA also notes its relevance in monitoring high-risk DTC patients or those with aggressive tumour subtypes, as it can inform further surgical or therapeutic decisions.

### 9.3. Alignment with ETA Guidelines

The ETA supports the ATA’s position and emphasizes the utility of FDG PET/CT in advanced or RAI-refractory DTC cases, highlighting its potential prognostic role, especially in aggressive subtypes and recurrence monitoring.

In RAI-refractory DTC, FDG PET/CT is particularly valuable for assessing tumour burden and monitoring disease progression.

The ETA advocates using Tg doubling time as a predictive marker to identify patients who may benefit from FDG PET/CT.

### 9.4. Controversies

Despite this guidance, several key controversies persist regarding the role of FDG PET/CT:

### 9.5. Initial Staging Debate

There is ongoing debate about the use of FDG PET/CT for initial staging and its prognostic value. While some studies suggest that PET/CT can identify aggressive or dedifferentiated DTC, both the ATA and ETA caution against its routine use in initial assessments, highlighting that its benefits may be limited to select cases.

### 9.6. Radiation Exposure and Cost

Concerns regarding radiation exposure and cost-effectiveness, particularly for low-risk patients, contribute to hesitancy surrounding the routine application of FDG PET/CT.

### 9.7. Prognostic Utility

While some studies have correlated metabolic parameters from PET/CT (e.g., SUVmax) with clinical outcomes, a consensus on its prognostic significance is lacking. Both the ATA and ETA acknowledge the potential utility of PET/CT in high-risk or radioiodine-refractory scenarios but warn against over-reliance without complementary biomarkers, such as Tg kinetics.

In summary, both the ATA and ETA endorse the use of FDG PET/CT in differentiated thyroid cancer for high-risk recurrence, cases with negative RAI scans, and specific aggressive subtypes. They recommend a case-by-case approach rather than routine application and call for further research to elucidate the roles of new tracers and predictive markers.

## 10. Conclusions

PET/CT is being increasingly recognized as a valuable tool in managing differentiated thyroid cancer, particularly in detecting recurrent or metastatic disease in patients with non-iodine-avid tumours or elevated Tg levels. While it is not typically part of the initial diagnostic process, its role in staging, recurrence detection, and monitoring treatment response is expanding. Future developments in novel radiotracers and theragnostic applications may further enhance the utility of PET/CT in thyroid cancer, offering the potential for more personalized and effective treatment strategies. However, challenges related to cost, availability, and standardization must be addressed to fully realize the potential of PET/CT in routine clinical practice.

## Figures and Tables

**Table 1:** PET/CT Applications in Differentiated Thyroid Cancer.

Stage of DTC	PET/CT Role
Initial Diagnosis	Limited role, mainly for incidental thyroid nodules
Staging	Supplementary to conventional imaging in selected cases (e.g., non-iodine-avid tumours)
Recurrence Detection	Valuable for identifying recurrent or metastatic disease, especially in patients with elevated Tg and negative RAI scans
Follow-up	Essential for monitoring patients with high-risk DTC, particularly those with aggressive subtypes or persistent disease

## Data Availability

No new data were created or analyzed in this study. Data sharing is not applicable to this article.

## Supplementary Materials

The following supporting information can be downloaded at: https://www.mdpi.com/article/10.3390/jcm13226918/s1, Table S1: Supplementary Tool for Recurrence Detection.

## Abbreviations

AJCC	American Joint Committee on Cancer
ATA	American Thyroid Association
DTC	Differentiated Thyroid Cancer
FDG	18F-Fluorodeoxyglucose
FNAB	Fine-Needle Aspiration Biopsy
FAP	Fibroblast Activation Protein
FTC	Follicular Thyroid Carcinoma
LNR	Lymph node ratio
MIP	Maximum Intensity Projection
NIS	Sodium Iodide Symporter
PET/CT	Positron Emission Tomography/Computed Tomography
PET/MRI	Positron Emission Tomography/Magnetic Resonance Imaging
PRRT	Peptide Receptor Radionuclide Therapy
PTC	Papillary Thyroid Carcinoma
PTMC	Papillary Thyroid Microcarcinoma
PSMA	Prostate-specific Membrane Antigen
RAI	Radioactive Iodine
RAIR-DTC	Radioactive Iodine-Refractory Differentiated Thyroid Cancer
SPECT	Single Photon Emission Computed Tomography
SUV	Standardized Uptake Value
Tg	Thyroglobulin
Tg-DT	Thyroglobulin-Doubling time
TKIs	Tyrosine Kinase Inhibitors
WHO	World Health Organization
